# Students in the Sex Industry: Motivations, Feelings, Risks, and Judgments

**DOI:** 10.3389/fpsyg.2021.586235

**Published:** 2021-02-25

**Authors:** Felicitas Ernst, Nina Romanczuk-Seiferth, Stephan Köhler, Till Amelung, Felix Betzler

**Affiliations:** ^1^Charité – Universitätsmedizin Berlin, corporate member of Freie Universität Berlin, Humboldt-Universität zu Berlin, and Berlin Institute of Health, Department of Psychiatry and Psychotherapie (CCM), Berlin, Germany; ^2^Charité – Universitätsmedizin Berlin, corporate member of Freie Universität Berlin, Humboldt-Universität zu Berlin, Berlin Institute of Health, Institute of Sexology and Sexual Medicine, Berlin, Germany

**Keywords:** sex work, students, stigma, motivations, feelings, prostitution

## Abstract

Student sex work is a current phenomenon all over the world, increasingly reported by the media in recent years. However, student sex work remains under-researched in Germany and is lacking direct first-hand reports from the people involved. Further, sex work remains stigmatized, and therefore, students practicing it could be at risk of social isolation and emotional or physical danger. Therefore, this study examines students working in the sex industry focusing on their personal experiences and attitudes toward them. An online questionnaire was completed by 4386 students from Berlin universities. Students who identified themselves as sex workers (*n* = 227) were questioned with respect to their motivations to enter the sex industry, characteristics of their job, feelings after the intercourse, and perceived risks. Student non-sex workers (*n* = 2998) were questioned regarding knowledge of and attitudes toward student sex workers. Most student sex workers reported that they entered the sex industry due to financial reasons (35.7%). The majority reported offering services involving direct sexual intercourse. Disclosing their job to friends, family, or others was associated with less problems with social isolation and in romantic relationships. With a total of 22.9%, student non-sex workers reported never having heard about students working in the sex industry. The most frequent emotions mentioned by them with regard to student sex workers were compassion and dismay (48.9%). There was no difference in happiness between student sex workers and non-sex working students. Through this research, it becomes evident that there are similarities between the student’s motivations to enter the sex industry, their feelings, and the problems they have to face. Moreover, prejudices still prevail about the life of student sex workers. Increasing understanding of student sex work might help those sex workers to live a less stigmatized life and thereby to make use of support from others. The universities as institutions could form the basis for this, e.g., by openly supporting student sex workers. This could help to encourage the rights of student sex workers and to gain perspective with respect to the sex industry.

## Introduction

Sex work is one of the oldest known professions present across different countries. A commonly used definition of “sex work” is the process of offering a sexual act for money or material goods (Comte, 2010). As written by Sagar et al. (2015a), in its broader definition, it may include indirect sexual exchange, such as lap dance and striptease, while in its narrower definition, it refers to direct sexual intercourse only. Several types of sex work are differentiated in the literature with respect to the type of execution such as “indoor” vs. “street” prostitution (Morton et al., 2012) or “voluntary” and “forced” sex work (Tyler and Johnson, 2006); however, literature focusing on forced or survival sex and sex trafficking should be differentiated from sex work carried out for economic purposes (Deshpande and Nour, 2013). While these differentiations have helped in the struggle for the de-stigmatization of sex work, their implied dichotomy may fail the realities of sex workers (Sandy, 2006).

In comparison to sex work in general, student sex work remains under-researched in Germany, and reports of first-hand experiences from the people directly involved are lacking. Student sex work is a widespread phenomenon all over the world (Roberts et al., 2007, 2010, 2013; Betzler et al., 2015; Sagar et al., 2015a). Roberts et al. (2013) studied the prevalence of student sex work in the United Kingdom and estimated that around 6% of all university students are working in the sex industry. Research by Sagar et al. (2015a) suggests that almost 5% of students in the United Kingdom are involved in sex work. Betzler et al. (2015) found that approximately 7% of the students in Berlin, Germany, are working or have been working in the sex industry. While some people are aware of the existence of student sex work and may even have considered entering the sex industry themselves, others are unaware of it (Roberts et al., 2010; Betzler et al., 2015). Until now, only a few studies have concentrated on students’ motivations to enter the sex industry (Roberts et al., 2010; Sagar et al., 2015a). These studies were carried out in countries that demand relatively high student fees and the primary motivation of the sex workers was to fund their education. In 2012, a project in the United Kingdom was launched to support student sex workers and to improve other people’s understanding of sex work/ers (Sagar et al., 2015a). The most frequent motivation stated by the students to enter the sex industry was to fund a particular lifestyle (63.5%). Flexible working hours were also an important factor, and another motivation was the feeling of pleasure while engaging therein. Students feared stigmatization, and as a result, their biggest concern was keeping their job a secret. The project also found that the surveyed universities avoided the topic of student sex work presumably due to fear of reputational damage. The project shows that the phenomenon of student sex work is often tabooed in society (Sagar et al., 2015b). This makes it not only hard for students engaged in the sex industry to openly talk about their fears and other feelings and make use of any support, but it also means that students involved in the sex industry may have to face severe stigmatization and prejudice (Sagar et al., 2015a). Therefore, addressing the characteristics of the job, the motivations to engage therein, and the feelings of the student sex workers is important as it provides a better understanding of student sex workers and the support services that they require.

Sex, as a subject of study, has long been a complex matter. Biological, evolutionary, cultural, political, and psychological influences have consistently contributed to evolving societal views toward those engaging in different forms of sexual practices (Best and Williams, 1997; Shackelford et al., 2015; Lucas and Fox, 2020). For example, sex work has often been a target of stigmatization (Goffman, 1986): sex work was associated with pathologic conditions, drug addiction, and sexually transmitted diseases in the past (Vanwesenbeeck, 2001; Comte, 2010). The stigmatization and criminalization of sex work are attributed to a long history, in particular focusing on patriarchal norms of society regarding especially female sexuality (Armstrong, 2019). It is believed that its function was mainly to exercise social control over women not complying to existing gender norms and thereby to regulate sexual competition (Levey and Pinsky, 2015; Rubin, 1992).

The relation between stigmatization and legislation is well established. Many states criminalized the profession, which leads to stigmatized attitudes and biased views toward sex workers (Sanders, 2016; Armstrong, 2019). In Germany, prostitution was regarded as an immoral profession by law until 2002, due to the meaning of §138 Abs. 1 “Bürgerliches Gesetzbuch” (BGB). Therefore, “prostitutes” had no legal claim for proper consideration. The Prostitution Act entered into force on January 1, 2002. Its objective was to improve the legal and social situations of sex workers (Deutscher Bundestag, Drucksache 14/5958). Today, sex work is at least partially legally regulated and considered as a profession in many Western countries, which in part leads to an increasing acceptance and understanding of the job (Immordino and Russo, 2015). However, in more than half of the countries of the world, sex work is still not fully legalized (Minichiello et al., 2018). For example, in some countries, the so-called Nordic Model was established, which defines selling sex as legal but buying it as illegal in order to protect the women involved (Vuolajärvi, 2019). Furthermore, many stigmas remain and sex workers are often seen as victims, forced to sell their bodies (O’Connell Davidson, 1995). Regarding student sex work, few studies reflected students’ knowledge of student sex workers and their view of the work (Roberts et al., 2007, 2010; Long et al., 2012). Previous studies focused on prevalence and demographic details of students working in the sex industry (Roberts et al., 2013; Betzler et al., 2015). However, studies focusing on the knowledge of and attitudes toward student sex workers are lacking (Roberts et al., 2007, 2010; Long et al., 2012). Examining this issue is important in order to understand how student sex workers may be received by their peers and how students’ attitudes influence the way sex workers see themselves in terms of self-stigmatization and related challenges and charges. Attitudes toward sex work in general are diverse and depend on variables such as “pro-feminist” attitudes or social desirability (Basow and Campanile, 1990; Long et al., 2012). In the study by Long et al. (2012) for example, results indicated that social desirability was associated with “stereotypical attitudes” about sex workers as, e.g., seeing sex workers as responsible for spreading sexual transmitted diseases. “Pro-feminist” attitudes were associated with seeing sex work as exploitation and oppression of women (Basow and Campanile, 1990).

In addition to stigma, post-traumatic stress disorder, depression, and other mental health problems, mostly caused by violence, are associated with working in the sex industry (Farley and Barkan, 1998; Chudakov et al., 2002). Possible psychological burden and distress within today’s sex workers may in part result from stigmas rather than from the work itself (Vanwesenbeeck, 2001), and it has been shown that stigmatization often plays an important role in the life of student sex workers (Sagar et al., 2015a). Most studies ascertaining these associations focused on street prostitution or trafficked women, whereas studies focusing on sex work in a broader sense did not find such correlations, i.e., Romans et al. (2001). The authors found no differences in mental health and self-esteem between female sex workers and age-matched women. These opposing results illustrate the importance of the distinction between sex trafficking and different types of sex work and the circumstances under which the sex work takes place. Therefore, this study also aims to focus on emotional well-being and any potential risks among the group of student sex workers in Berlin.

The study at hand is both one of the first studies examining students working in the sex industry in Berlin and that includes first-hand experiences from the people directly involved. It firstly concentrates on the characteristics, motivations, feelings of, and risks for students working in the sex industry. Secondly, the paper examines students’ knowledge about and attitudes toward student sex workers focusing on feelings the job provokes in students who are not engaged in sex work. Addressing these research aims in the context of the university environment is important as both lack of awareness of student sex work and specific attitudes toward the student sex workers may result in sex working students to work in secret or to become a higher risk of discrimination, harassment, and bullying from their peers compared to non-sex working students (Wong et al., 2011; Armstrong, 2019). Furthermore, it is critical to improve the awareness of the universities and the authorities in charge, so that they are able to offer specific support to student sex workers in Berlin and elsewhere.

## Materials and Methods

### Participants

The analysis was conducted in the form of a cross-sectional study based on data collected by Betzler et al. (2015). A two-part online questionnaire was distributed among students studying at four of Berlin’s major universities; 4423 students completed the questionnaire. A total of 37 participants did not correctly fill in the questionnaire and had to be excluded. The final sample consisted of 4386 students; mean age was 24.4 years (*SD* = 3.7). With respect to the gender, 44.1% of the participants were female and 32.1% were male; 13.6% did not specify their gender. The research comprised students undertaking their bachelor’s, master’s degree, as well as Ph.D. students. Subjects of study were divided into groups as proposed by the Federal Statistical Office of Wiesbaden (Statistisches Bundesamt Wiesbaden [SBW], 2015^1^). The group of subjects with the largest proportion of students was “Law, economics and social sciences”. A total of 227 out of 4386 participants stated being or having been involved in the sex industry. Eighty-nine of the student sex workers who specified their gender were female and 61 were male, χ^2^ (1, *N* = 150) = 0.004, *p* = 0.95; mean age was 25.3 (*SD* = 4.2). The baseline characteristics are summarized in Table 1. Participation was voluntary and not compensated. All of the participants were German-speaking as the questionnaire was delivered in German.

**TABLE 1:** Baseline characteristics for the group of student sex workers and “other” students.

	**Sex workers**	**Non-sex working students**
**Characteristics**	**% (*n*)**	**% (*n*)**
Mean age (*SD*)	25.3 (*SD* = 4.2)	24.4 (*SD* = 3.7)
Gender		
Female	39.2 (89)	38.5 (1603)
Male	26.9 (61)	26.7 (1110)
Missing values	33.9 (77)	34.8 (1446)
Nationality		
German	56.8 (129)	58.8 (2447)
European	3.1 (7)	3.6 (150)
Others	1.3 (3)	1.3 (56)
Missing values	38.8 (88)	36.2 (1506)
Faith/religion		
Christianity	20.7 (47)	25.6 (1064)
Judaism	1.8 (4)	0.2 (10)
Islam	0.9 (2)	0.8 (32)
Atheism	29.5 (67)	30.6 (1274)
Agnosticisms	5.3 (12)	5.6 (232)
Others	5.3 (12)	1.8 (75)
Missing values	36.6 (83)	35.4 (1472)
Subjects of study		
Law, economics, or	11.0 (25)	21.2 (635)
social sciences		
Human sciences	11.0 (25)	12.5 (374)
Engineering	7.5 (17)	9.6 (289)
Maths or sciences	6.6 (15)	11.6 (348)
Medicine or health sciences	5.3 (12)	12.3 (370)
Others	4.9 (11)	6.5 (196)
More than one subject	4.4 (10)	5.3 (158)
Agronomy, forestry,	2.6 (6)	1.6 (49)
veterinary medicine, or		
nutrition science		
Arts	1.8 (4)	1.4 (41)
Sports	0.9 (2)	0.3 (9)
Missing values	44.0 (100)	17.7 (529)

### Instruments and Procedure

The two-part online questionnaire designed by Betzler et al. (2015) was laid out as follows: the first part, which all participants completed, contained items on sociodemographic data (e.g., age, gender, nationality, faith/religion, and subject of study). The second part applied either to student sex workers or to non-sex working students. If students reported being or having been engaged in the sex industry, they were asked about the characteristics of their job (services offered, frequency of appointments with clients, the places they meet, and paying conditions), their motivation for having entered the sex industry, and risks of their work (problems they might face, experience of violence). At the end of the questionnaire, students who had any kind of experiences related to sex work were invited to contact the study team if they felt the need to talk about their experiences. Furthermore, their feelings after the intercourse and happiness in the last 3 months were assessed. Hybrid questions with multiple responses were mostly used. Motivation was examined using item sets. For happiness, the scale from the socioeconomic panel (SOEP) was used (seven-point Likert scale). This study defines sex work in the broader sense. Students offering any type of sex work such as prostitution in the narrow sense, escort services with or without sexual intercourse, striptease, and webcam or phone sex were included. Participants could provide additional types of sex work.

Students not involved in the sex industry were asked in the second part about their awareness of (if and how they know student sex workers) and attitudes toward student sex workers (feelings students have when thinking about student sex workers and problems they assume student sex workers have to face). Since attitudes determine stigmatization (Jonsson and Jakobsson, 2017), these items provide insights on this issue. Lastly, their happiness in the last 3 months was investigated. Likewise, hybrid questions with multiple responses and a seven-point Likert scale for happiness were used to examine the research questions.

The questionnaire was distributed via mailing lists among students of Berlin’s major universities. The participants were instructed on the first page to truthfully and completely fill in the questionnaire. Informed consent was obtained from the participants. The questionnaire was completed independently through the online platform “SoSci Survey”.

### Data Preparation and Analyzes

In the initial study by Betzler et al. (2015), a plausibility filter was used to exclude participants who did not correctly fill in the questionnaire. In addition to this, maximum values for interval scaled new variables (number of friends the student sex workers disclose the job to and clients per night, per week, and per day) were specified; values exceeding their respective thresholds were labeled as missing values. The data were analyzed using SPSS version 23. Explorative analysis and Kolmogorov–Smirnov tests were conducted to gain an insight into the data and to ensure normal distribution.

With respect to the first research aim, descriptive statistics were applied in order to examine the answers given on questions concerning the characteristics of the job, the motivations of the students, disclosing the job to someone else, the feelings, and the experience of violence. To investigate potential effects of disclosing the job to someone else and to examine differences within the group of student sex workers with respect to gender and happiness, Mann–Whitney *U* tests and cross-tabulations were calculated.

With respect to the second research aim, descriptive statistics were applied to investigate the awareness of students working in the sex industry. Further, descriptive statistics were used to examine problems the sex workers face and non-sex working students assume they have. Cross-tabulations were calculated to explore the feelings students have when thinking of student sex workers. Finally, a Mann–Whitney *U* test was used to examine differences in happiness of both groups. The value α was set to 0.05.

## Results

### Characteristics of Student Sex Work

The most frequent service students provided was found to be prostitution in the narrow sense (21.6%; *n* = 49). Escort services including sexual intercourse were offered by 18.5% (*n* = 42) and 9.7% (*n* = 22) stated offering escort services excluding sexual intercourse. Striptease or web cam services were provided by 11.0% (*n* = 25). A total of 9.7% (*n* = 22) of students stated providing other services, such as erotic massages or working as a porn actor. One third did not specify the service they offered (33.5%; *n* = 76); the students were allowed to give multiple answers. No difference in happiness could be found between sex workers offering sexual intercourse and those not offering it (*z* = -0.64, *p* = 0.53).

The mean of the number of clients student sex workers met per night was 2.9 (*SD* = 4.5); per week, 4.0 (*SD* = 4.9); and per month, 9.6 (*SD* = 28.3). The most frequent places of appointment were a hotel or the apartment of the client (hotel: 22.0%, *n* = 50; apartment of the client: 19.8%, *n* = 45); 10.6% (*n* = 24) reported having the appointments in a public place, 6.2% (*n* = 14) in a car, and 4.8% (*n* = 11) in a brothel. Other places were reported by 10.6% of the students; among others, massage parlors and the apartment of the student sex worker were mentioned. The question was not answered by 43.2% (*n* = 98); it was possible to give multiple responses.

The students stated obtaining between 15€ and 600€ per working hour (*M* = 113.58, *SD* = 96.73); this is slightly less than the average earnings per service (*M* = 142.87, *SD* = 134.08). An amount of 1.3% (*n* = 3) reported not getting paid with money but with, e.g., food, cigarettes, a photo-shoot, or a sleeping place.

### Motivations to Enter the Sex Industry

**FIGURE 1:**
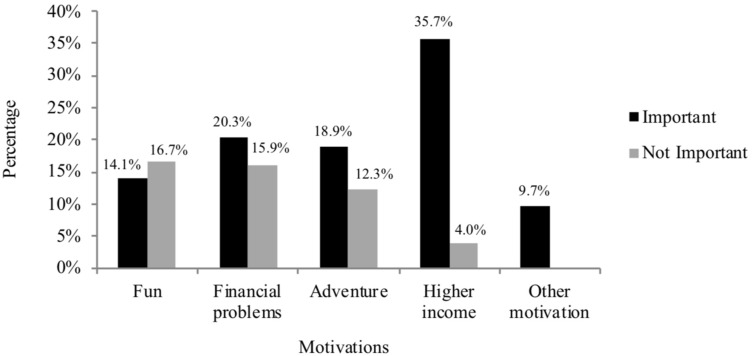
Percentage of motivations students reported being important or not important.

To investigate the motivations to enter the sex industry, the answers “not important at all” and “not important”, as well as the answers “important” and “very important” have been pooled. Motivational categories were fun, financial problems, adventure, and higher income. Additional motivations could be provided by participants. Among others, self-affirmation, curiosity, and flexible working hours were mentioned. Results are shown in Figure 1.

### Disclosing the Job to Someone Else

Not disclosing the job to someone else was reported by 9.7% (*n* = 22) of the students; 52.9% (*n* = 120) stated having told someone about it. The question was not answered by 37.4% (*n* = 85). Having talked to the partner about the job was reported by 23.8% (*n* = 36) of the students, who specified who they have talked to. Only 7.9% (*n* = 12) stated having talked to their family about it. Most students stated that they have told their friends about their job (37.7%, *n* = 57); the mean number of friends they talked to was 6.34 (*SD* = 8.85). Some students also stated being in contact with other student sex workers (15.3%, *n* = 22); the average amount of sex workers they were in contact with was 2.96 (*SD* = 1.86). The students were allowed to give multiple responses.

A non-significant trend was found of student sex workers who disclosed their job to someone else being happier than those who did not disclose it (*z* = -1.88, *p* = 0.06). Furthermore, disclosing the job to someone else was related to problems the students reported to face: χ^2^ tests revealed that there is a difference between sex workers who disclose their job or not with respect to the problems reportedly faced. Students who stated not having disclosed their job to someone else report problems with social isolation [(33.6% vs. 76.2%), χ^2^ (1, *N* = 128) = 13.17, *p* = 0.001] and in the partnership [(36.4% vs. 76.2%), χ^2^ (1, *N* = 128) = 11.31, *p* = 0.001] more often. No differences were found with respect to physical violence, mental stress, lowered self-esteem, persecution by the police, and health risks. The results are summarized in Table 2.

**TABLE 2:** Results of chi-square tests regarding the relationship between disclosing the job to someone else and perceived problems of the job.

**Problems**	**Total (*n*)**	**Disclosing the job (%)**	**Not disclosing the job (%)**	**Chi-square tests of independence**
Social isolation	128	33.6	76.2	χ^2^ (1) = 13.17 *p* = 0.001*
Problems in partnership	128	36.4	76.2	χ^2^ (1) = 11.31, *p* = 0.001*
Physical violence	128	17.8	28.6	χ^2^ (1) = 1.31, *p* = 0.25
Mental stress	128	39.3	61.9	χ^2^ (1) = 3.68, *p* = 0.06
Lowered self-esteem	128	16.8	33.3	χ^2^ (1) = 3.05, *p* = 0.13
Persecution by the police	128	13.1	23.8	χ^2^ (1) = 1.60, *p* = 0.31
Health risks	128	39.3	52.4	χ^2^ (1) = 1.25, *p* = 0.26

*p < 0.05.

### Experiences of the Work

Most students that offer sexual intercourse stated that the feelings after the intercourse depend on the client (18.5%, *n* = 42). Feeling good after the intercourse was reported by 3.5% (*n* = 8). A total of 6.2% (*n* = 14) reported that the intercourse does not influence their feelings and 1.3% (*n* = 3) stated not feeling good after it. Feeling something else was reported by 2.6% (*n* = 6); mentioned were *inter alia* shame or pride. With a total of 10.6% (*n* = 24), the students stated not to offer sexual intercourse (e.g., telephone sex); 57.3% (*n* = 130) did not answer this question.

Most students stated not having experienced violence while conducting their job (37.0%, *n* = 84). A total of 5.7% (*n* = 13) stated having experienced violence and the rest did not answer this question. The types of violence students referenced included both physical violence from clients and verbal abuse from others. χ^2^ tests revealed no difference in experiencing violence with respect to the service students offered; i.e., no difference was found between selling direct vs. indirect sexual exchange (10.7% vs. 14.5%), χ^2^ (1, *N* = 90) = 0.24, *p* = 0.62. In line with these findings, 43.6% (*n* = 99) stated being protected by someone while conducting their job; less student sex workers reported not being protected by anyone (15.9%, *n* = 36). The question was not answered by 40.5% (*n* = 92). Most students who specified by whom they are protected stated friends knew the place they were working at (22.2%, *n* = 30). With a total of 8.9% (*n* = 12) students reported being protected by working in a brothel, 3.7% (*n* = 5) reported working under a procurer’s protection. Some sex workers stated being protected in a different way, e.g., partner or an agency knew the places they were working at (11.9%, *n* = 16).

### Gender Differences

Differences found regarding gender were that female sex workers stated meeting more clients who are in a relationship than males [(79.4% vs. 46.7%), χ^2^ (1, *N* = 49) = 5.25, *p* = 0.04]. Also, females disclosed their job more often to friends than male sex workers [(58.3% vs. 36.8%), χ^2^ (1, *N* = 110) = 4.60, *p* = 0.03]. No difference between female and male sex workers was found in the services they offer, in the motivations to enter the sex industry, in experiencing violence, or in being protected. Neither male nor female sex workers were found to have a higher happiness score (*z* = -0.1.31; *p* = 0.19).

### Awareness of and Attitudes Toward Students Working in the Sex Industry

Most of the students who did not state an engagement in the sex industry reported knowledge about this phenomenon through media (62.2%, *n* = 1866). A total of 18.8% (*n* = 565) reported they heard about it in conversations, 1.7% (*n* = 52) stated knowing student sex workers since they have been clients. A few students reported knowing a student sex worker privately (4.2%, *n* = 127). A total of 22.9% (*n* = 686) of the students reported never having heard about the phenomenon of students working in the sex industry. It was possible to give multiple answers.

The most frequent problem respondents assume student sex workers have to face was mental distress. This was, alongside problems in the partnership, also the problem stated or assumed most frequently by student sex workers. Only 0.9% (*n* = 2) of the student sex workers and 0.3% (*n* = 9) of the non-sex working students stated not to have or not to assume any problems. Descriptive statistics are reported in Table 3 (multiple answers enabled).

**TABLE 3:** Descriptive statistics of potential problems sex workers are assumed to face.

	**Sex workers (*n* = 227)**	**Non-sex working students (*n* = 2998)**
**Problems**	**% (*n*)**	**% (*n*)**
Problems in partnership	24.2 (55)	54.5 (1634)
Mental stress	24.2 (55)	63.5 (1904)
Health risks	23.3 (53)	61.8 (1852)
Social isolation	22.9 (52)	43.6 (1306)
Physical violence	11.0 (25)	44.0 (1318)
Lowered self-esteem	11.0 (25)	47.2 (1416)
Persecution by the police	8.4 (19)	9.7 (290)
Others	3.5 (8)	4.3 (129)
None	0.9 (2)	0.3 (9)

*Note: This question was posed to both students who stated to be engaged in sex work and to those who did not. Hence, it does reflect the assumption of problems stated by non-sex workers as opposed to both assumptions and actually experienced problems by sex workers. This constellation does not allow for direct statistical comparison. It also explains higher values, e.g., for physical violence stated here vs. actually experienced physical violence assessed by a separate question (11.0% vs. 5.7%).*

The most frequent feelings students mentioned having while thinking of students working in the sex industry were *compassion and dismay* (48.9%, *n* = 1465), followed by *curiosity* (40.9%, *n* = 1227). Other students stated *lack of understanding* (19.0%, *n* = 571), *respect* (11.9%, *n* = 358), and *contempt* (4.2%, *n* = 126). Other feelings, such as *disconcertment*, *regret*, *astonishment*, *helplessness*, and *disgust* were reported by 10.0% (*n* = 301). The students were allowed to give multiple responses. Feelings students have when hearing about student sex workers were linked to the knowledge of students working in the sex industry and lack thereof: students not knowing this phenomenon reported *lack of understanding* more commonly than students knowing it [(19.7% vs. 27.4%), χ^2^ (1, *N* = 2666) = 16.73, *p* = 0.001] and less often the feeling of *respect* [(14.3% vs. 10.3%), χ^2^ (1, *N* = 2667) = 6.63, *p* = 0.01] as well as *curiosity* [(47.2% vs. 41.6%), χ^2^ (1, *N* = 2666) = 6.67, *p* = 0.01].

There was no difference in happiness during the last 3 months between student sex workers and non-sex working students (*z* = -0.79, *p* = 0.43); the median of happiness in both student sex workers and non-sex working students in the last 3 months was 8.0 (IQR = 2).

## Discussion

The aims of this exploratory study were (a) to gain a first insight into primary motivations driving students in Berlin, a major German metropolitan area, to enter the sex industry and to examine characteristics of their work, their feelings, and risks connected to the work; and (b) to investigate judgments and attitudes by non-sex working students toward student sex workers.

### Motivations to Enter the Sex Industry

The most frequent services offered by students working in the sex industry were prostitution in the narrow sense, meaning sexual intercourse for money (21.6%) and escort services including sexual intercourse (18.5%). Previous studies, however, reported that student sex workers more often offer services excluding rather than including direct sexual–monetary exchange (Roberts et al., 2013; Sagar et al., 2015a). As these studies by Roberts et al. (2013) and Sagar et al. (2015a) were conducted in countries demanding relatively high student fees, the different results are surprising. Since in Berlin student loans do not exist to this extent, one could assume that the students would not have to improve their financial situation by means of sex work. In this study, most sex workers (35.7%) stated that their primary motivation to enter the sex industry was indeed the possibility to obtain a higher income than in other jobs; only 4.0% stated that this was not important for them. A few (20.3%) opted for financial hardship as primary motivation, while nearly the same amount (15.9%) stated that this was not important. Financial hardship might therefore not be the only reason to enter the sex industry. Other reasons might include the flexibility of the job, as students are often not able to work regularly due to their studies. This is in line with the finding of Jenkins (2006), who also ascertained that financial hardship was not always the most relevant motivation for students to enter the sex industry. It was rather the fact of gaining higher income in a more flexible way than in other jobs. However, there are some students who stated that they entered the sex industry due to a critical financial situation.

Research by Sagar et al. (2015a) stated that both female as well as male students are working in the sex industry; sex work was even found to be more common among men than women. The current study did not find differences in gender within the student sex workers. Additionally, the study showed no difference between female and male sex workers with respect to several variables: no difference was found, among others, in the services they offer, in the motivations to enter the sex industry, or in experiencing violence. Regarding experiencing stigma, this study took a more generalized approach and did not take into account the differences between people’s experiences (e.g., with respect to gender or ethnicity), which would be of interest for future studies.

### Frequency and Payment

Regarding the appointments with clients and the payment student sex workers receive, the results found in this study were similar to previous studies (Kontula, 2008; Sagar et al., 2015a). Sagar et al. (2015a) reported that most students (54.1%) work less than five hours per week and 51.3% earn less than €300 per month.

### Disclosing Work to Others

Most students stated that they chose to disclose their job to friends or to their partner, which mirrors findings from Roberts et al. (2013). Only a few student sex workers stated not having disclosed their job to someone else; consequences were increased problems in their partnerships and increased social isolation. Furthermore, there was a tendency of student sex workers who disclosed their job to someone else being happier than those who did not disclose their job. Previous studies showed that student sex workers suffer most when they feel like they are not able to talk to anyone about their profession (Sagar et al., 2015a). This might be a consequence of the taboo and the stigmatization of sex work. A previous study examining sex workers in Hong Kong showed that stigma can have several negative effects on mental as well as physical health (Wong et al., 2011): this includes direct forms of bullying, physical and verbal violence, as well as rather indirect forms through which the sex workers feel the need to isolate themselves socially and fear utilizing the help available (e.g., health services). As seen in the study at hand, there are several problems that sex workers have to face. However, lowering the taboo of the profession and being able to talk openly about it might help to reduce some of these problems, such as social isolation, mental stress, and health risks (Wong et al., 2011; Armstrong, 2019).

### Experiences of the Work

In our study, 37% of the students working in the sex industry did not experience violence while conducting their job; only 5.7% stated having experienced violence. This might be due to the fact that many student sex workers (43.6%) stated being protected by someone while conducting their job. These results are in line with the finding reported by Sagar et al. (2015a) that only a minority of student sex workers reported a lack of safety in the job; 75.5% stated to feel safe very often or always while conducting their job. Despite this, there are some students who stated to have experienced violence to which one should pay attention. The types of violence students referenced included both physical violence from clients as well as verbal abuse from others. It is assumed that removing the stigma around student sex work may help to make the profession safer (Armstrong, 2019). Previous studies showed that violence perpetrated against sex workers is often a consequence of stereotypical and hostile views toward sex workers (Sanders, 2016). Challenging such negative stereotypes and stigmatization may therefore help to build respect for sex workers and, in doing so, work toward reducing violence.

For both questions regarding feelings and experience with violence during sex work, there was a high prevalence of missing values. These were notably higher than for other parts of the questionnaire, which could be due to these questions being perceived as particularly sensitive and the students felt intimidated or ashamed to answer the question through an online questionnaire. As a result, there may have been a bias toward individuals with less negative feelings toward their work. Future studies could attempt a more personal interview approach in order to gain a greater understanding of these factors.

### Students’ Views Toward Sex Workers and Differences Between Groups

Roberts et al. (2010) reported in their study that the majority of the students (58.5%) are aware of students working in the sex industry. This study found similar results. Over 62% of the non-sex working students reported to be aware of it through the media. Nonetheless, there is still a substantial number of students (22.9%) who reported never having heard about the phenomenon before. Research by Long et al. (2012) showed that people who know someone working in the sex industry have more positive views toward sex workers than others. This study supports these findings. Students who have never been confronted with student sex workers prior to the study reported more often the feeling *lack of understanding* when thinking of student sex workers than students who were aware of the phenomenon. Moreover, they reported less often the feelings *respect* and *curiosity* than students who were aware of students working in the sex industry. These findings point out the importance of increasing the awareness of student sex work. Research shows that there is still limited acceptance of sex work as a profession (Long et al., 2012; Ma et al., 2018). Despite its legalization, many prejudices exist concerning sex work (Sagar et al., 2015a). In the past, sex workers were often seen as culprits, spreading sexually transmitted diseases (Vanwesenbeeck, 2001). Even until now, sex workers in general are seen as victims, which experience several problems. The fact that the majority of the students answered that they felt compassion and dismay while thinking of student sex workers emphasizes this observation. Regarding happiness, however, the current study showed that there was no difference in happiness with respect to working in the sex industry or not.

### Limitations

Despite the fact that there was a total of 4386 participants, students who are engaged in the sex industry or who know someone engaged in it may have been more willing to fill in the questionnaire, leading to a sample bias. The study was conducted in a metropolitan city, which might have led to a distorted higher number of sex workers, even though other studies found similar prevalence. On the other hand, it is conceivable that there is a substantial number of unreported cases of sex working students who did not want to provide such intimate information in an online questionnaire, even though it was clearly stated that data were collected anonymously. Further, the study did not include a response option for transgender and non-binary identity, which would be important to include in future studies as such individuals make up a notable percentage of sex workers (Fitzgerald et al., 2015).

A self-report instrument was used to collect the data. Participants might not always tell the truth, particularly concerning a sensitive topic like this. In addition to quantitative research methods, other studies adopted qualitative methods such as interviews (Jenkins, 2006; Sagar et al., 2015a). Qualitative research methods might raise the expressiveness of the answers; at the same time, it might be more accessible for participants to answer questions on this topic in privacy.

The questionnaire used for this analysis was not validated. This is partly due to the fact that there is a lack of validated instruments for the study of sex work and in particular of sex work stigma, which should be dealt with in further research.

The question on “feelings after the intercourse” was displayed rather broadly in the questionnaire (e.g., lacking a comparison to either not having intercourse with a client or not having intercourse at all) and thus may have been more open to interpretation from the respondents. Therefore, the answers given on this question have to be interpreted with caution.

In this work, we derived the parameter of stigmatization mainly from the question about the feelings of non-sex working students toward sex workers, as we believe stigma (only partially dependent on the degree of perception of sex workers themselves) is mainly formed this way (Jonsson and Jakobsson, 2017). We asked the question of conceivable negative experiences to both sex workers and non-sex workers at the same time, which provides a certain comparison (presented in Table 3). However, this question involved various kinds of negative experiences, some of which only serve as a vague estimation for experiencing stigma (e.g., social isolation).

Lastly, the study at hand contains a relatively high number of missing data. As the aim of the research was to get an extensive insight into students working in the sex industry through consulting as many individuals as possible, all given answers from incomplete questionnaires were included. Also, the missing data might reflect the sensitivity of the topic and the results of the study should not be generalized. As mentioned above, in the future, the use of qualitative in addition to quantitative methods could be considered to gain a better understanding on some of the more sensitive topics surrounding student sex work.

## Conclusion

This research provides further insights into the topic of student sex work, as it is the first study in Germany focusing on both student sex workers and non-sex working students as well as comparing these two “groups”. With respect to previous studies, it can be seen that there are similarities to data from other countries in what motivates students to enter the sex industry, what student sex workers feel, and which problems they have to face. Despite a relatively well working system of financial support, a number of German students are nonetheless working in the sex industry. Further data from other German areas (smaller metropolitan or even non-metropolitan areas) need to be collected to complete this picture. As mentioned above, stigmatization of sex work is attributed to a long history. While it is acceptable to sell most things, selling sexual services is still met with a lot of prejudice. Nowadays, a primary argument against sex work is the violence sex workers are exposed to during their work. However, the violence may, to some extent, be the result of stigmatization and negative stereotypes (Armstrong, 2019), which should be addressed. Sex work has been a legal profession in Germany since 2002, and while this legalization has already contributed to a better understanding of the industry, sex workers still face stigma, which can result in hostility, exclusion, and violence. While the impact of the legal situation could only be ascertained through comparative studies with other countries, knowledge about student sex workers was associated with more positive and less negative feelings toward student sex workers. Such shift in public perception will help student sex workers to live a less stigmatized life, talk to someone else about their job, and make use of any support, without being afraid of condemnation. The universities as institutions could form the basis for this, e.g., by openly supporting student sex workers. For example, in the United Kingdom, The University of Leicester published a toolkit for their staff in order to fight discrimination (“Student Sex Work Toolkit for Staff in Higher Education” by Trueman et al., 2020). This may help to encourage the rights of student sex workers, gain perspective with respect to the sex industry, and, at the same time, provide support for the students involved in the sex industry.

## Data Availability Statement

The raw data supporting the conclusions of this article will be made available by the authors, without undue reservation upon request.

## Ethics Statement

Ethical review and approval was not required for the study on human participants in accordance with the local legislation and institutional requirements. The patients/participants provided their written informed consent to participate in this study.

## Author Contributions

FB was responsible for the conceptualization, design of the questionnaire, data acquisition, and supervision. FE was responsible for the data analysis. FE, FB, and NR-S were responsible for the writing—draft preparation. FB, NR-S, SK, and TA were responsible for the writing—review and editing. All authors contributed to the article and approved the submitted version.

## Conflict of Interest

The authors declare that the research was conducted in the absence of any commercial or financial relationships that could be construed as a potential conflict of interest.

## Footnotes

1 https://www.destatis.de/DE/Methoden/Klassifikationen/BildungKultur/StudentenPruefungsstatistik.pdf?__blob=publicationFile
